# Indium Tin Oxide-Free Inverted Organic Photovoltaics Using Laser-Induced Forward Transfer Silver Nanoparticle Embedded Metal Grids

**DOI:** 10.1021/acsaelm.2c00217

**Published:** 2022-06-02

**Authors:** Sergey M. Pozov, Kostas Andritsos, Ioannis Theodorakos, Efthymios Georgiou, Apostolos Ioakeimidis, Ayala Kabla, Semyon Melamed, Fernando de la Vega, Ioanna Zergioti, Stelios A. Choulis

**Affiliations:** † Molecular Electronics and Photonics Research Unit, Department of Mechanical Engineering and Materials Science and Engineering, Cyprus University of Technology, Limassol 3603, Cyprus; ‡ School of Applied Mathematical and Physical Sciences, National Technical University of Athens, Iroon Polytechniou 9, Athens 15780, Greece; § PV Nano Cell, 8 Hamasger St., Migdal HaEmek 2310102, Israel

**Keywords:** laser-induced forward transfer, Ag nanoparticle ink, ITO-free electrodes, metal grids, inverted organic photovoltaics, reverse nanoimprinting processing, charge selective contacts, printed electronics

## Abstract

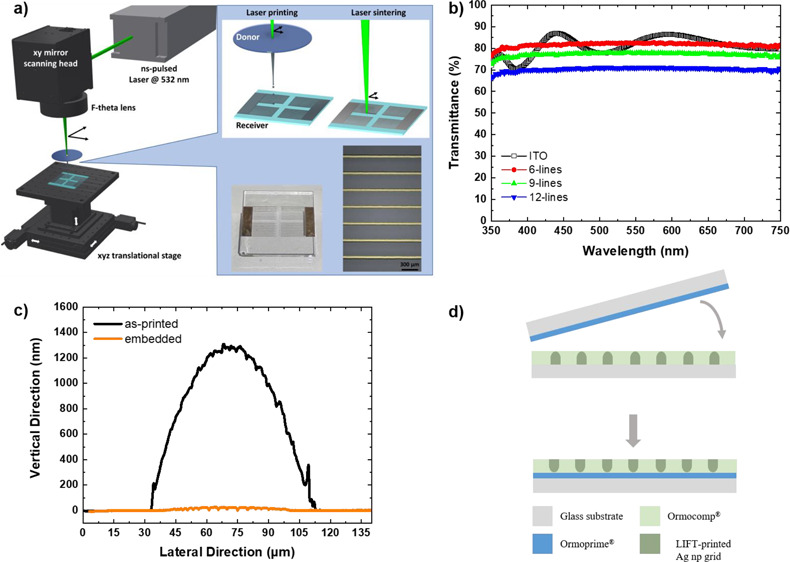

Laser-induced forward transfer (LIFT) printing has emerged as a valid digital printing technique capable of transferring and printing a wide range of electronic materials. In this paper, we present for the first time LIFT printing as a method to fabricate silver (Ag) nanoparticle (np) grids for the development of indium tin oxide (ITO)-free inverted PM6:Y6 nonfullerene acceptor organic photovoltaics (OPVs). Limitations of the direct use of LIFT-printed Ag np grids in inverted ITO-free OPVs are addressed through a Ag grid embedding process. The embedded laser-printed Ag grid lines have high electrical conductivity, while the Ag metal grid transparency is varied by altering the number of Ag grid lines within the inverted OPVs’ ITO-free bottom electrode. Following the presented Ag-grid embedding (EMP) process, metal-grid design optimizations, and device engineering methods incorporating an EMB-nine-line Ag np grid/PH500/AI4083/ZnO bottom electrode, we have demonstrated inverted ITO-free OPVs incorporating laser-printed Ag grids with 11.0% power conversion efficiency.

## 1 Introduction

In the past few years, organic photovoltaics (OPVs) have achieved tremendous power conversion efficiency improvements mainly due to the emergence of n-type organic semiconductor nonfullerene acceptors (NFAs). In comparison to the traditional fullerene acceptors, the NFAs have lower cost due to simpler synthetic methods, suitable band gap that increases the active-layer absorption, and tunable energy levels that favor the open-circuit voltage.^1,2^ Organic solar cells based on NFA have boosted the OPVs’ power conversion efficiency from approximately 10 to over 17%,^3−6^ while a significant improvement in device stability has been also reported in the literature.^7−9^ Possibilities of the new organic electronic material systems in large-scale OPV manufacturing have also been recently investigated, and successful upscaling modules have been fabricated.^10,11^ Aiming toward commercialization, the widely used transparent indium tin oxide (ITO) conductive electrode increases the overall cost, while its brittleness makes it incompatible to flexible optoelectronics and large-scale roll-to-roll manufacturing. Among the proposed solutions, the inkjet-printed metal nanoparticle ink is the most used method for the development of printed ITO-free electrodes for organic optoelectronic applications. Highly efficient ITO-free OPVs,^12−14^ perovskite PVs,^15^ and OLED^16,17^ devices have been successfully fabricated using silver and copper nanoparticle inks, while possibilities of up-scalability have also been investigated.^18^ Despite the successful and promising results, inkjet printing requires specific ink formulation properties (surface tension, viscosity, etc.) to avoid nozzle clogging and control flow.^19^ On the other hand, the laser-induced forward transfer (LIFT) digital printing technique provides more flexibility on the donor ink properties.^20^ LIFT printing was introduced by Bohandy and his co-workers,^21^ and its working principle usually employs a pulsed laser source that provides the energy to a laser transparent substrate that can be coated with a solid, paste, or liquid donor layer, which absorbs the laser energy and transfers the material toward the acceptor substrate. In case of liquid donors, the absorption of pulsed laser results in the formation of a high-pressure bubble, which expands and creates a stable jet of the donor material. Details of the LIFT technology can be found in review papers.^20,22^ Laser printing has been successfully applied in organic electronics and biosensors^23^ and demonstrated to be a vital digital printing technique capable of transferring and printing a wide range of materials and systems such as metal nanowires,^24,25^ metal nanoparticles,^26^ conducting polymers,^27^ and light-emitting polymer diode pixels.^28^ Optimization studies of laser printing and laser sintering of silver nanoparticle (Ag-np) inks on glass^29−34^ and flexible substrates^35−37^ were performed in terms of laser parameters, sacrificial layer properties, and rheological properties of the inks. LPKF provides LIFT integrated systems in the market^38^ and shows the potentials of the digital laser transfer printing technology for a range of applications. Main electronic applications of laser-printed metal np patterns include conduction paths in sensors^39^ and metallization methods in the flexible electronic industry.^40^ The LIFT technology has not been previously applied for the development of ITO-free organic optoelectronic applications.

Motivated by the compatibility with various inks, scalability, and high productivity rates, LIFT-printed and laser-sintered Ag np grids are implemented for the first time as an alternative to the ITO electrode in inverted OPV devices. The highly efficient binary organic NFA-based semiconductor material system consisting of the PBDB-T-2F (PM6) polymer donor and the BTP-4F (Y6) as the nonfullerene acceptor is used as an active layer in the inverted OPV device structure.^41^ The presented study is focused on the Ag np grids as an ITO-free bottom electrode, with the aim to identify the optimized processing conditions and device structure engineering to achieve highly efficient LIFT-printed inverted ITO-free OPVs. Different PEDOT:PSS formulations were investigated in this work relevant to their potential to planarize the as-laser-printed (not-embedded) metal grid height. We show that PEDOT:PSS HIL-E100 provided high-quality thick buffer layers (∼700 nm), which however were not enough to planarize effectively the ∼1300 nm metal-grid height on glass substrates. As a result, limitations on the performance of not-embedded Ag-grid based inverted OPVs on glass substrates were identified. Not-embedded LIFT-printed Ag np grid ITO-free OPVs exhibited a very low shunt resistance resulting to ITO-free OPVs with low reliability and limited maximum power conversion efficiency (PCE) of 3.7%.

We demonstrate that the reverse nanoimprinting embedding method is necessary for the planarization of the Ag np grids to achieve functional ITO-free OPV devices. The effect of PEDOT:PSS in an embedded (EMB) Ag grid/PEDOT:PSS/ZnO bottom electrode structure is investigated using different PEDOT:PSS formulations. PH500 is identified as the optimum PEDOT:PSS for the laser-printed embedded Ag-grids. The conductivity of laser-printed metal grids is in the range of 40,000 S/cm, while transparency of the metal grid was varied by varying the number of ∼70 μm width metal grid lines incorporated in the design of the ITO-free bottom electrode. The experimental results show that a nine-line metal grid design provides the optimum balance between conductivity and transparency for the laser-printed Ag nanoparticle current collecting bottom electrode grid. The ITO-free inverted OPVs with the EMB-nine-line Ag np grid/PH500/ZnO bottom electrode exhibited a maximum PCE of 8.9%, while the reference ITO-based devices provided a PCE of 12.1%. Further device optimization is achieved by incorporating a bilayer PEDOT:PSS configuration within the device structure, with laser-printed ITO-free inverted OPVs incorporating the EMB-nine-line Ag np grid/PH500/AI4083/ZnO bottom electrode demonstrating a maximum PCE of 11.0%.

## 2 Results and Discussion

### 2.1 LIFT-Printed and Laser-Sintered Ag Np Grids

**Figure 1:**
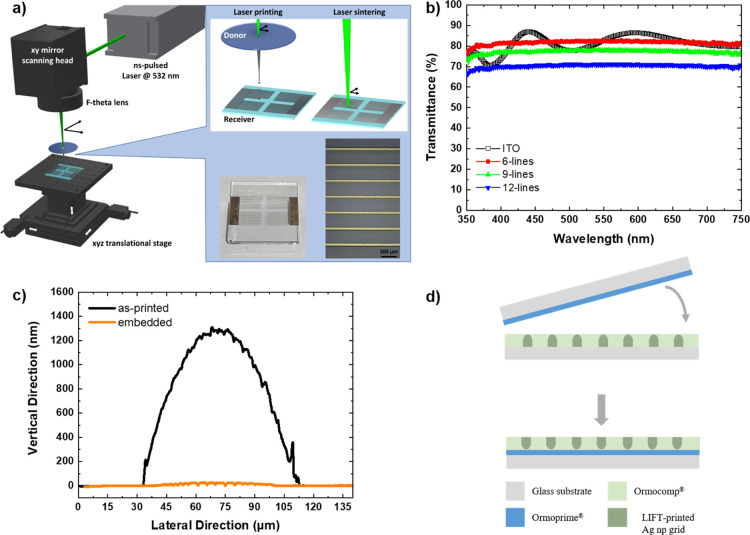
(a) Schematic illustration of the laser configuration and the laser-printing and laser-sintering process. In the inset, two examples of laser-printed and -sintered samples are presented. (b) Optical transmission spectra of parallel lines assembling 6-, 9-, and 12-line grids. (c) Schematic explanation of the reverse nanoimprinting process for the embedding of Ag grids using Ormocomp and Ormoprime resins. (d) Cross-section profiles of the as-laser-printed versus embedded laser-printed Ag np grids.

**Figure 2:**
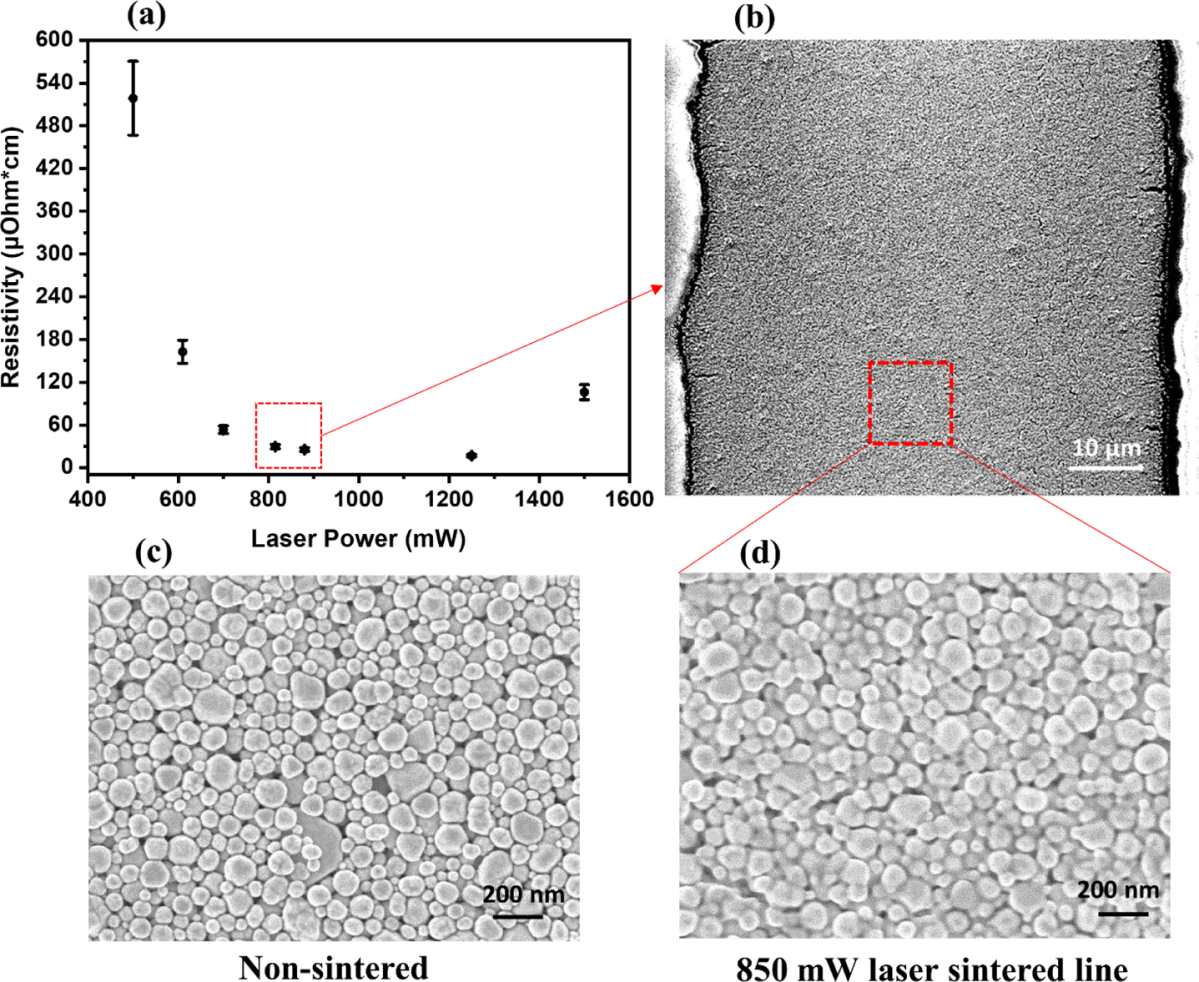
Laser sintering of LIFT-printed Ag nps: (a) resistivity to laser power correlation, (b) SEM image of laser-sintered Ag np line, (c) magnified SEM of nonsintered Ag nps, and (d) magnified SEM image of 850 mW laser-sintered Ag nps.

A schematic illustration of the laser-printing and -sintering process is presented in Figure 1a. A nanosecond (ns) pulsed laser at 532 nm and high repetition rate (up to 500 kHz) create a laser beam that is guided through mirrors and lenses toward a galvanometric set of two mirrors (galvo) with configurable speed, thus enabling the two-dimensional scanning of a quartz donor that is coated with a Ag np ink (Sicrys I70DB-H72_E32) at the liquid phase developed by PV Nano Cell. The Ag np ink contains 70% Ag in diethylene glycol monobutyl ether (DGBE) and has a viscosity of 630 cP (0.63 N·s/m^2^), surface tension of 28.2 dyn/cm (2.82 × 10^–6^ N·m), and particle size in the range of 30–150 nm. Utilizing a blade coater, the quartz donor is coated with the aforementioned ink resulting in a 12–14 μm thickness film. The investigation for the determination of the laser printing parameters and the ink’s viscosity influence is thoroughly presented in ref (33). The same study was performed while taking into consideration the new ink’s viscosity as well as the surface properties. Therefore, the donor is irradiated with a laser fluence of 125 mJ/cm^2^ and beam size of 35 μm, resulting in a dimensionally controlled printed droplet. Associating a galvo speed of 0.45 m/s with a laser repetition rate of 10 kHz and keeping the distance between donor and receiver at 50 μm, a drop on demand deposition was achieved, succeeding in the formation of smooth lines with no bulging effects and discontinuities. Subsequently, the same laser configuration was employed for the selective laser sintering of the Ag np grid. For the decision on the laser sintering parameters, an investigation concerning the laser repetition rate, the laser beam size, and the scanning speed was performed, as in ref (35). For this soda-lime glass substrate and ink, the optimum laser sintering power was determined after a series of tests correlating the laser power with the resulting resistivity as presented in Figure 2a. The resistivity values display a significant drop with the increase of the laser power, reaching a plateau after 700 mW. For higher laser powers, the printed lines exhibited cracks and delamination effects that resulted in an electrical performance drop. Thus, 850 mW laser power was selected for the sintering of the Ag np printed lines to minimize the undesired crack effects that are limited at the thinner edges of the line as presented in scanning electron microscopy (SEM) images in Figure 2b. At the same time, this specific laser power value induced the necessary nanoparticle’s necking (Figure 2d) compared to the nonsintered nanoparticles (Figure 2c), enabling the printed lines to obtain a resistivity value of less than 30 μΩ·cm. By employing a 60 kHz repetition rate combined with a 0.1 m/s galvo scanning speed and a laser spot of 100 μm beam size, a spatial overlap of 98% was realized. The resulted electrical properties of laser-printed and -sintered Ag np grid on soda-lime glass were determined using four points on the side-bar and the grid lines (grid layout Figure 1a). The measured sheet resistance in the case of the side-bar was 0.08 Ω/sq, which according to the measured thickness results in a resistivity of 25 μΩ·cm and conductivity of ∼40,000 S/cm. As expected, similar values were calculated on the grid lines, with a sheet resistance of 0.21 Ω/sq, resistivity of 27 μΩ·cm, and conductivity of ∼37,000 S/cm. The achieved electrical conductivity values are much higher in comparison to our reference ITO electrode with a sheet resistance of 5 Ω/sq, resistivity of 30 μΩ·cm, and conductivity of ∼6600 S/cm.

To form the ITO-free bottom electrode, a parallel line pattern connected to busbars was laser-printed on the soda-lime glass substrate to prepare electrodes for four device pixels (9 mm^2^) as presented in the inset photograph of Figure 1a. The specific grid pattern was chosen due to its simplicity, fast printability, and high reproducibility, while 6-, 9-, and 12-line Ag np grids were printed to include a range of 15–30% grid coverage area. The transparency evaluation of the different printed electrodes is presented in Figure 1b. The average transmittance of different grid patterns in the visible spectrum were calculated as ∼82% for 6-line, ∼77% for 9-line, and ∼70% for 12-line grids. Compared to the ITO average transmittance of ∼81%, 9-line and 12-line grids show lower transmittance due to increased shadowing effects. However, by incorporating a higher number of LIFT-printed Ag np lines, the resistance measured from side-bar to side-bar was reduced from 2.7 to 2.0 to 1.5 Ω at the respective number of the 6-, 9-, and 12-line grid. A detailed study on the influence of optical and electrical properties of the laser-printed Ag np grid on the OPV device performance is presented later. Geometrical characteristics of the laser-printed Ag np lines were analyzed using cross-section profilometry as presented in Figure 1c. When analyzing the geometrical characteristics of the printed Ag np grids in Figure 1c, **∼**70 μm line width and ∼1300 nm line height were recorded. Even though the relatively narrow width favors the optical transmittance of the metal-grid based electrode, the grid height presented limitations for direct use within the OPV device structure. Organic optoelectronic devices consist of very thin layers. Therefore, such high laser-printed Ag np line height (∼1300 nm) based ITO-free bottom electrodes have a great possibility to penetrate through the functional overcoated layers and contact the top electrode, leading to short circuit devices. To avoid the above-mentioned limitation, a thick PEDOT:PSS layer can be used to overcoat and planarize the surface of the metal grids.^42^ To examine that approach, ITO-free devices were fabricated using the laser-printed Ag np grids overcoated with a ∼700 nm thick PEDOT:PSS HIL-E100 formulation. The specific formulation is developed by Heraeus to be used on rough surfaces with the ability to planarize and keep high transparency even at very thick films. However, even in the case of the ∼700 nm thick PEDOT:PSS, the device performance was very poor and reliability issues were observed due to the penetration of the laser-printed Ag np grid through the functional layers creating a short circuit with the top electrode. The best performing ITO-free inverted OPVs using non-embedding as-laser-printed Ag np grids/HIL-E100 (∼700 nm) provided a PCE of only 3.7%, while the PCE of the respective reference ITO/HIL-E100 (∼700 nm) device was 10.6%, as presented in Figure S1a and Table S1 (Supporting Information). The performance of the presented inverted OPV devices using the ∼700 nm thick HIL-E100 PEDOT:PSS is much lower compared to the 13.5% PCE obtained for the ITO/ZnO/PM6:Y6/MoO_3_/Ag-based devices. The reduced short-circuit current (*J*_sc_) and fill factor (FF) in ITO-based solar cell devices are mainly attributed to the thick HIL-E100 layer, while significantly lower photovoltaic parameters of ITO-free devices strongly depend on the lack of Ag np grid planarization that promoted high leakage currents and device short circuits (Figure S1). Our ongoing efforts aim to maintain similar values of Ag grid resistance with reduced Ag-grid height to simplify processing steps for the development of high-performance ITO-free inverted OPVs. To avoid grid penetration and device failure in the presented work, Ag grid total planarization was achieved using the highly effective reverse nanoimprinting method.^12,14,18,43^ The planarization is performed using highly transparent resins, a sacrificial glass substrate containing the as-printed and sintered Ag np grid, and the final glass substrate where the embedded (EMB) grid is transferred, as schematically described in Figure 1d. As presented in Figure 1c, Ag np grids were successfully embedded and planarized, maintaining their electrical and optical properties. Atomic force microscopy (AFM) investigation of the surface topography before and after the embedding process is shown in Figure S2 (Supporting information). The root-mean-square (rms) roughness and peak-to-valley values of the printed and sintered Ag np grid surface were reduced from 16.8 to 1.7 nm and from 140 to 24.1 nm, respectively, after the embedding process (Figure S2 a,b), indicating the effectiveness of electrode planarization by the presented embedding process.

### 2.2 Effect of PEDOT:PSS Formulation on the Performance of ITO-Free Inv-OPVs

**Figure 3:**
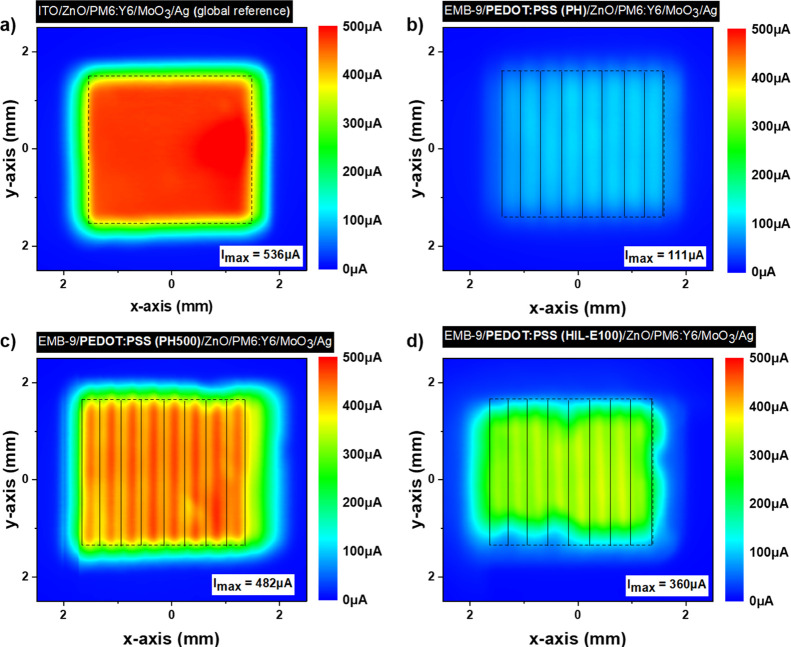
Photocurrent mapping images of 9 mm^2^ inverted OPV devices: (a) ITO-based reference device and ITO-free devices using the EMB nine-line Ag np grid with different PEDOT:PSS formulations, (b) PH, (c) PH500, and (d) HIL-E100. The reference device in that case was ITO/ZnO/PM6:Y6/MoO_3_/Ag.

For the development of ITO-free inverted OPVs, LIFT-printed and embedded Ag np grids were implemented with the approach of using a PEDOT:PSS/ZnO electron selective contact for the bottom electrode of the OPV device structure, as was previously reported.^42^ As also indicated in more details above, for the completion of inverted OPV devices, PM6 as a polymer donor and Y6 as a nonfullerene molecular acceptor were used within the active layer, with thermally evaporated MoO_3_ as the hole transporting layer and Ag as the top contact. Three different PEDOT:PSS (PH, HIL-E100, and PH500) formulations were selected based on their intrinsic electrical conductivity and employed in the following bottom electrode configuration EMB-nine-line Ag np grid/PEDOT:PSS/ZnO with the aim to investigate the functionality of the laser-printed Ag np grid ITO-free electrode within the reported inverted OPV device structure. The photocurrent (PCT) mapping characterization presented in Figure 3 shows the generated photocurrent images after a constant wavelength laser beam is absorbed by the OPV device and current is extracted by the apparatus. PCT images of ITO-free OPV devices with different PEDOT:PSS formulations are presented and compared to our ITO-based global reference device with the following configuration: ITO/ZnO/PM6:Y6/MoO_3_/Ag. Parallel line grids with nine lines and 21% grid surface coverage area calculated from the geometrical characteristics of the lines were selected to be used as the constant grid pattern in the study of the PEDOT:PSS formulation effect. Among the ITO-free devices, a homogeneous photocurrent distribution and the highest absolute photocurrent were detected in the case of the PH500 formulation, with a value of 482 μΑ that was very close to the reference ITO-based device with 536 μΑ. Significantly lower absolute photocurrent values were recorded in the case of HIL-E100 (360 μΑ) and PH (111 μΑ). The reduced PCT can be directly correlated to the intrinsic electrical conductivity of each PEDOT:PSS formulation. The electrical conductivity of the corresponding PEDOT:PSS formulation was evaluated through four-point probe measurements, and the values for each formulation were ∼0.10 S cm^–1^ for PH, ∼60 S cm^–1^ HIL-E100, and ∼500 S cm^–1^ for PH500. Illuminated *J*–*V* characteristics and photovoltaic parameters of the fabricated devices with different PEDOT:PSS formulations were also investigated as presented in Figure S3 and Table S2 (Supporting Information). All the devices presented the same open-circuit voltage (*V*_oc_) of 0.76 V; however, significant differences were presented in *J*_sc_ and FF*.* The EMB-Ag np grid/PH device showed the lowest *J*_sc_ (7.5 mA/cm^2^) due to its lowest intrinsic electrical conductivity, which significantly increased the series resistance of the device and therefore reduced FF (34.3%), resulting in a PCE of 1.9%. On the other hand, although HIL-E100 demonstrated a relatively high *J*_sc_ (16.8 mA/cm^2^), the high series resistance of the HIL-E100 based devices induces a low FF of 37.1% as can be seen in the *J*–*V* characteristics presented within Figure S3, providing therefore a PCE of 4.7%. Eventually, the best performance was provided by the PEDOT:PSS (PH500) formulation. By incorporation of PH500 on top of the LIFT-printed Ag np grid, the highest *J*_sc_ of 22.5 mA/cm^2^ and the lowest series resistance among the investigated formulations were achieved, resulting in increased FF of 52.5% and PCE of 8.9%. The presence of a suitable PEDOT:PSS buffer layer on top of the embedded Ag np grid is therefore an improving strategy for the development of high-performance ITO-free inverted OPVs using LIFT nanoparticle-based metal grids.

### 2.3 Effect of Ag Np Grid Surface Coverage Area on the Performance of ITO-Free Inv-OPVs

**Figure 4:**
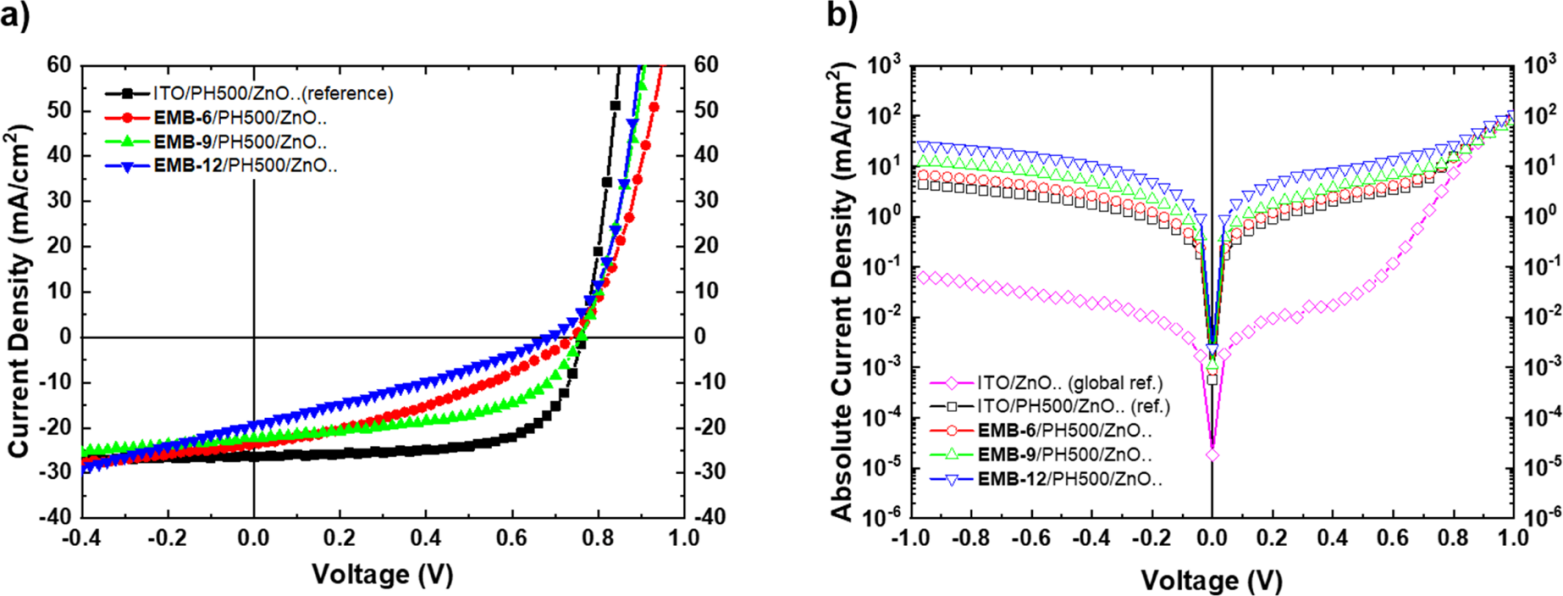
Inverted OPVs with different bottom electrodes, ITO reference, and ITO-free embedded Ag np grids with different numbers of grid lines. (a) Illuminated *J*–*V* characteristics and (b) dark *J*–*V* characteristics.

To define the ideal balance between optical transparency and electrical conductivity of laser-printed and embedded Ag np grids, it is necessary to determine the ideal grid coverage area. Metal grid coverage areas have been previously defined to be in the range of ∼17% for normal structure OPVs^14^ and ∼23% for OLEDs.^18^ In general, the optimum metal grid coverage area strongly depends on the requirements of the optoelectronic device and the metal grid properties such as deposition technique, material, design pattern, and geometrical characteristics. Since LIFT-printed Ag np grids are for the first time incorporated in ITO-free inverted OPVs, the optimum balance between the grid electrical conductivity and transparency was necessarily investigated using 6-, 9-, and 12-line grids, with corresponding surface coverage areas of ∼14, ∼21, and ∼28%. The different grids were laser-printed, embedded, and incorporated in the bottom ITO-free electrode structure EMB-Ag np grid/PH500/ZnO. The corresponding *J*–*V* characteristics of the devices are shown in Figure 4, while the photovoltaic parameters of the best devices are presented in Table 1. Significant differences were presented in the *J*–*V* characteristics especially in FF of the ITO-free devices compared to the ITO-based reference device that in this case was ITO/PH500/ZnO/PM6:Y6/MoO_3_/Ag. Dark *J*–*V* characteristics (Figure 4b) revealed the highest leakage current in both reverse and forward bias regimes for the 12-line embedded Ag np grid followed by the 9-line grid, the 6-line grid, and finally the reference ITO/PH500/ZnO bottom electrode device. A similar trend was also presented in the shunt resistance (*R*_sh_) calculated from the dark *J*–*V* plot as the inverse slope around *J* (0 mW/cm^2^, 0 V). The 12-line grid exhibited the lowest *R*_sh_ followed by the 9-line grid, 6-line grid, and ITO-based reference device as presented in Table 1.

**Table 1:** Photovoltaic Parameters of the Best Inverted OPV Devices with Laser-Printed and Embedded Ag Np Grids/PEDOT:PSS (PH500) and Different Numbers of Grid Lines^a^

bottom electrode	*V*_oc_ [V]	*J*_sc_ [mA/cm^2^]	FF [%]	PCE [%]	*R*_s_ [Ω·cm^2^]	*R*_sh_ [Ω·cm^2^]
ITO/PH500/ZnO..(ref. device)	0.76	26.8	59.6	11.7 ± 0.3 (12.1)	1.72	217.5
EMB-6/PH500/ZnO..	0.76	23.3	41.4	5.8 ± 0.8 (7.3)	4.76	163.0
EMB-9/PH500/ZnO..	0.76	22.5	52.5	8.4 ± 0.5 (8.9)	2.02	121.5
EMB-12/PH500/ZnO..	0.70	19.6	31.5	3.9 ± 0.5 (4.6)	1.93	45.0

a Average PCE values obtained from several experimental device runs are presented together with standard deviations and maximum values in brackets.

Leakage current is commonly affected by several factors, such as substrate cleaning, electrode interlayers, film thickness, and film deposition techniques. In this work, such a large variation in the leakage current is attributed mainly to the intrinsic properties of the PEDOT:PSS PH500 and the morphology of the functional layers on top of embedded Ag np grids. A significantly higher leakage current was also recorded in the ITO-based device with the ITO/PH500/ZnO electrode in comparison to the ITO/ZnO without the PH500 interlayer. The high electrical conductivity of the PH500 formulation is attributed to the lower PSS to PEDOT ratio that however affects negatively its charge carrier blocking properties, creating intrinsic leakage currents.^44^ Thereby, the presence of PH500 itself significantly increases the leakage current of the device. It has been shown that leakage or shunt current can lead to significant decreases in both the *V*_oc_ and FF of organic solar cells.^45^ The additional impact on leakage characteristics can be attributed to the morphology of the functional layers and interfaces. The embedded and flattened Ag np grid lines rise to ∼40 nm after PEDOT:PSS PH500 coating and annealing, as presented in Figure S4b. The increase of Ag np grid line height can be attributed to the thermal expansion and contraction due to the first thermal annealing step of PEDOT:PSS PH500. Therefore, the increased number of printed Ag np grid lines increases the possibility of morphology abnormalities and leakage currents that negatively affect the device’s FF values, as presented in Figure 4b and Table 1. Affected by the highest leakage current and lowest optical transmittance, the 12-line Ag np grid exhibited the lowest photovoltaic efficiency parameters among the investigated metal-grid designs, resulting to a maximum PCE of 4.6%. The photocurrent mapping analysis shown in Figure S5 (Supporting Information) presents the homogeneous current distribution of the well-defined solar cell active region for the inverted ITO-free OPVs using PEDOT:PSS PH500/ZnO as the electron selective contact and Ag np grid with different numbers of parallel lines. The highest absolute photocurrent was recorded for the 6-line grid with 506 μA followed by the 9-line (482 μA) and 12-line (440 μΑ) grids, presenting a similar trend with the *J*_sc_ values as shown in Table 2. The higher generated photocurrent for the six-line grid is attributed to its highest optical transmittance due to the reduced shadow losses, as presented in Figure 1b. Since PCT analysis is also affected by the functional layer morphology and interfaces, differences in PCT images between 6-, 9-, and 12-line grids may be presented according to embedded line ″rise″ that was discussed earlier. The six-line metal grid provided higher *R*_sh_ of 163 Ω·cm^2^ and *J*_sc_ of 23.3 mA/cm^2^ in comparison to the nine-line grid with 121.5 Ω·cm^2^ and 22.5 mA/cm^2^, respectively. The maximum PCE of 7.3% for the six-line grid is significantly lower than 8.9% for the nine-line grid device. The reason is the much lower FF of the six-line grid (41.4%) in comparison to 52.5% for the nine-line grid. The lower FF of the six-line grid is mainly due to the higher series resistance (*R*_s_), which is almost doubled (4.76 Ω·cm^2^) compared to the nine-line grid devices (2.02 Ω·cm^2^). Thus, it is inferred that by applying the six-line grid, the required conductivity could not be achieved for a well-functioning Ag np grid electrode. On the contrary, the 12-line metal grid showed the lowest *R*_s_ (1.93 Ω·cm^2^) due to the highest number of conductive paths. Thus, the impact of both high leakage current and excessive shadowing losses resulted in the lowest PCE. Therefore, it is concluded that the nine-line Ag np grid design with a ∼21% coverage area provides the optimum trade-off between transparency and conductivity for implementation of the bottom electrode in inverted ITO-free OPVs using LIFT Ag np embedded grids.

**Table 2:** Photovoltaic Parameters of the Optimized Inverted OPV Devices with Laser-Printed and Embedded Ag Np grids Using Bilayer PEDOT:PSS (PH500/AI4083) Layers^a^

bottom electrode	*V*_oc_ [V]	*J*_sc_ [mA/cm^2^]	FF [%]	PCE [%]	*R*_sh_ [Ω·cm^2^]
ITO/PH500/ZnO..	0.76	26.8	59.6	11.7 ± 0.3 (12.1)	217
ITO/PH500/AI4083/ZnO..	0.78	24.5	66.7	12.8 ± 0.5 (13.5)	232
EMB-9/PH500/ZnO..	0.76	22.5	52.5	8.4 ± 0.5 (8.9)	121
EMB-9/PH500/AI4083/ZnO..	0.78	21.2	60.5	10.4 ± 0.4 (11.0)	294

a Average PCE values obtained from several experimental device runs are presented together with standard deviations and maximum values in brackets.

### 2.4 PEDOT:PSS (PH500/AI4083) Bilayer to Further Improve the ITO-Free Inv-OPVs

**Figure 5:**
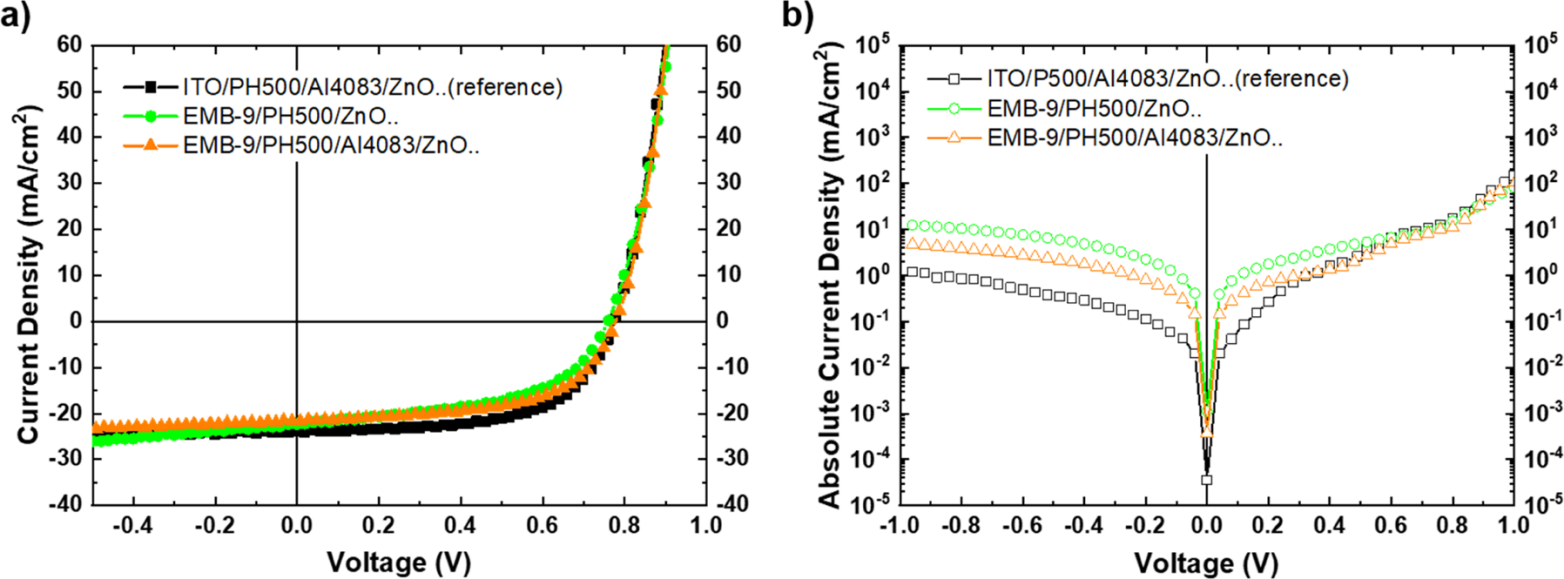
Inverted OPVs with different bottom electrodes, ITO reference, and ITO-free embedded nine-line Ag np grids using PEDOT:PSS (PH500) and a bilayer (PH500/AI4083). (a) Illuminated *J*–*V* characteristics and (b) dark *J*–*V* characteristics.

As a final device engineering method, the ITO-free inverted OPV device with the nine-line embedded Ag np grid and PH500/ZnO electron selective contact was further optimized by introducing an additional 25 nm thin layer of low conductive PEDOT:PSS (AI4083) on top of the PH500. The combination of two PEDOT:PSS formulations in a bilayer configuration (PH500/AI4083) is proposed as a method to improve the nine-line embedded Ag np grid/PH500/AI4083/ZnO bottom electrode’s electron selectivity and promote the further reduction of ITO-free OPV device leakage currents. For better understanding, the same bilayer PEDOT:PSS (PH500/AI4083) concept is applied on the reference ITO-based inverted OPV bottom electrode (ITO/PH500/AI4083/ZnO). Compared to PH500/ZnO, the incorporation of the PH500/AI4083/ZnO electron selective bottom contact within the inverted OPV bottom electrode in both ITO-based and ITO-free inverted OPVs only slightly affected the overall transmittance and device series resistance, therefore resulting in a small reduction of *J*_sc_ values as presented in Figure 5a and Table 2. Despite the slightly reduced *J*_sc_, a significant improvement was achieved in FF. The FF values increased from 59.6 to 66.7% in ITO-based inverted OPVs and from 52.6 to 60.5% in ITO-free inverted OPVs under investigation. Dark *J*–*V* characteristics presented in Figure 5b show the significant reduction in leakage current for the nine-line EMB Ag nps/PH500/AI4083/ZnO bottom electrode-based device in comparison to the nine-line EMB Ag nps/PH500/ZnO bottom electrode device. The reduced leakage current is ascribed to the higher shunt resistance values extracted from dark *J*–*V*’s, as shown in Table 2. Specifically, the shunt resistance in inverted ITO-free OPV devices increased from 121 Ω·cm^2^ for the nine-line EMB Ag nps/PH500/ZnO bottom electrode to 294 Ω·cm^2^ for the nine-line EMB Ag nps/PH500/AI4083/ZnO bottom electrode. Surface topography studies indicate that the rms-roughness was reduced from 20.3 nm in the case of Ag np grid/PH500 to 12.2 nm when AI4083 was deposited on top of PH500 (Ag nps/PH500/AI4083). The incorporation of AI4083 provides a smoother surface for the overcoated ZnO layer within the ITO-free inverted OPV bottom electrode (embedded Ag np grid/PH500/AI4083/ZnO), providing more intimate interfaces and thus reducing the device leakage currents. As a result, AI4083 provided a further increase in the performance of ITO-free OPVs with an average PCE of 10.4% (max PCE of 11.0%) and ITO-based OPVs with average an PCE of 12.8% (max PCE of 13.5%).

## 3 Conclusions

LIFT-printing and laser-sintering techniques were employed to develop Ag metal nanoparticle grids as an alternative to the bottom ITO electrode in inverted structure nonfullerene acceptor PM6:Y6 based OPVs. The developed Ag np grid lines exhibited a height of ∼1300 nm and width of ∼70 μm, while their electrical conductivity on glass substrates was in the range of 37–40 kS/cm. To avoid short-circuit solar cell device effects, the reverse nanoimprinting method was applied to embed and planarize the LIFT-printed Ag np metal grids. The incorporation of PH500 PEDOT:PSS within the LIFT-printed embedded Ag np grid/PH500/ZnO bottom electrode provided the best performing PEDOT:PSS formulation for the development of high-performance ITO-free inverted OPVs. To define the optimum laser-printed Ag grid surface coverage area for inverted ITO-free OPVs, different numbers of embedded LIFT-printed Ag nps-parallel lines (6, 9, and 12) were investigated. The nine-line LIFT-printed Ag np grid provided the optimum balance between laser-printed Ag grid transparency and conductivity. The corresponding inverted ITO-free OPV devices provided a maximum PCE of 8.9% due to limited FF values originated from high leakage currents. We show that the incorporation of low conductive AI4083 PEDOT:PSS within the laser-printed Ag nanoparticle-based metal-grid inverted ITO-free OPV bottom electrode (embedded Ag np grid/PH500/AI4083/ZnO) reduces leakage currents and improves charge carrier selectivity for the inverted ITO-free OPVs incorporating LIFT-printed Ag np grids. The optimized ITO-free inverted OPVs incorporating nine-line laser-printed Ag nanoparticle embedded metal grids (Ag grid/PH500/AI4083/ZnO/PM6:Y6/MoO3/Ag) demonstrated up to 11% PCE.

## 4 Experimental Methods

### 4.1 Materials

Pre-patterned indium tin oxide (ITO) (sheet resistance 4 Ω/sq) on a 1.5 × 1.5 cm soda-lime glass substrate was purchased from Psiotec Ltd. Clevios (P VP AI4083), (PH), (PH500) and (HIL-E100) PEDOT:PSS (3,4-ethylenedioxylthiophene):poly(styrene sulfonate) formulations were purchased from Heraeus. Zinc oxide (ZnO, 2.5 wt %) in a mixture of butanols (N-10-Flex) was provided by Avantama. The PM6 polymer donor and the BTP-4F (Y6) nonfullerene acceptor were purchased from Solarmer Energy, Inc. For the embedding and flattening of laser-printed Ag np grids, the reverse nanoimprinting transfer procedure was applied^12^ using Ormocomp and Ormoprime resins purchased from Micro Resist Technology. All the other chemicals were purchased from Sigma-Aldrich.

### 4.2 Device Fabrication

Reference ITO-based inverted OPV devices were fabricated according to the following device structure: ITO/ZnO/PM6:Y6/MoO_*x*_/Ag. ITO substrates were subsequently cleaned in acetone and isopropanol under sonication for 10 min and then dried and exposed to UV-ozone treatment for 5 min. The ZnO (∼20 nm) film was spin-coated at 4000 rpm (Delta 6RC-Suss MicroTec, Garching, Germany) and was thermally annealed at 120 °C for 20 min under ambient atmosphere. The polymer PM6:Y6 (D/A = 1:1.2, 16 mg mL^–1^ in total) was dissolved in chloroform (CF) with the solvent additive of 1-chloronaphthalene (CN) (0.5%, v/v) and spin-coated on ZnO at 3000 rpm to achieve a ∼130 nm thickness and then transferred into a nitrogen-filled glovebox for thermal annealing at 110 °C for 10 min. To complete the device stack, 10 nm (0.2 Å/s) of a molybdenum oxide (MoO_*x*_) anode interlayer and 80 nm (2 Å/s) silver (Ag) were thermally evaporated in a vacuum chamber (Angstrom Engineering, Kitchener, Canada) at a base pressure of ∼1 × 10^–6^ mbar through a shadow mask, resulting in a device active area of 9 mm^2^. In the case of ITO-free devices, Ag np grids were embedded and then cleaned with isopropanol. All PEDOT:PSS formulations were filtered through a 0.22 μm polyvinylidene difluoride (PVDF) filter prior to processing. Ethylene glycol (5% v/v) was added to PH500 formulation. PEDOT:PSS formulations were spin-coated to maintain the same layer thickness of ∼40 nm on both ITO and ITO-free electrodes. In case of the bilayer PH500/AI4083, AI4083 was spin-coated at 5000 rpm to give a thickness of ∼25 nm. PEDOT:PSS films were thermally annealed at 140 °C for 20 min under ambient atmosphere. The 2.5 wt % zinc oxide (ZnO) nanoparticle (size ∼10 nm) in the mixture of butanols was purchased from Avantama (N-10-Flex). The ZnO electron transporting layer (∼20 nm) was dynamically spin-coated at 5000 rpm (Delta 6RC-Suss MicroTec, Garching, Germany) and was thermally annealed at 120 °C for 20 min under ambient atmosphere. The rest of the layers was deposited as has been described before, and the final ITO-free OPV device stack was Ag np grid/PEDOT:PSS/ZnO/PM6:Y6/MoO_*x*_/Ag.

### 4.3 Characterization

For the film characterization, a Shimadzu UV-2700 UV–vis spectrophotometer was used to measure the optical transmittance of the different surface coverage area grids, while a Veeco Dektak 150 profilometer was used to define the metal grid profile and the thickness values of every functional layer of the OPV device. A four-point probe (Jandel RM3000) conductivity meter was employed for sheet resistance measurements. The characterization of complete devices was performed under ambient atmosphere without device encapsulation, while a 9 mm^2^ mask was used to ensure the exact active area of the device. The current–voltage characteristics were measured with a Keithley source measurement unit (SMU 2420) and a calibrated Newport solar simulator equipped with a Xe lamp (AM1.5G spectrum at 100 mW cm^–2^ as measured by an Oriel 91150V calibration cell equipped with a KG5 filter). Photocurrent mapping measurements were performed under 405 nm laser excitation wavelength, 50% laser intensity, and 40 μm laser spot size using a Botest PCT photocurrent system.^46^

## Supporting Information Available

Photovoltaic parameters of ITO and ITO-free OPVs using a thick PEDOT:PSS buffer layer; photovoltaic parameters of ITO-free OPVs using various PEDOT:PSS formulations; profilometric analysis of not-embedded Ag np grids and *J*–*V* characteristics of the corresponding solar cell devices; AFM images before and after the embedding process; profilometric analysis of the embedded grid; and photocurrent mapping images of ITO-free OPVs using different number of Ag grid lines (PDF)
