# Supplementary material for: Indium Tin Oxide-Free Inverted Organic Photovoltaics Using Laser-Induced Forward Transfer Silver Nanoparticle Embedded Metal Grids
Source: ACS Appl Electron Mater. 2022 Jun 2;4(6):2689–98. doi: 10.1021/acsaelm.2c00217 (PMC9245438; doi:10.1021/acsaelm.2c00217)
Supplement: Supplementary file 1 — el2c00217_si_001.pdf [file el2c00217_si_001.pdf]

Supporting Information

Indium Tin Oxide-Free Inverted Organic Photovoltaics using Laser Induced Forward Transfer Silver Nanoparticle Embedded Metal Grids

Sergey M. Pozov [1], Kostas Andritsos [2], Ioannis Theodorakos [2], Efthymios Georgiou [1], Apostolos Ioakeimidis [1], Ayala Kabla [3], Semyon Melamed [3], Fernando de la Vega [3], Ioanna Zergioti [2] and Stelios A. Choulis [1]*

*Corresponding Author: stelios.choulis@cut.ac.cy

[1] Molecular Electronics and Photonics Research Unit, Department of Mechanical Engineering and Materials Science and Engineering, Cyprus University of Technology, Limassol, 3603, Cyprus

[2] School of Applied Mathematical and Physical Sciences, National Technical University of Athens, Iroon Polytechniou 9, 15780, Athens, Greece

[3] PV Nano Cell, 8 Hamasger St., Migdal HaEmek, 2310102, Israel

Tables

Table S 1: Photovoltaic parameters of the best performed ITO-based and ITO-free inverted OPV devices using 700nm thick PEDOT:PSS HIL-E100 formulation. As laser-printed (not embedded) Ag nps 9-line grid was used.

Device	V_{oc} [V]	J_{sc} [mA/cm^2]	*FF* [%]	*PCE* [%]
ITO/ZnO/PM6:Y6/MoO_3/Ag (global reference)	0.80	24.2	69.6	13.5
ITO/HIL-E100/ZnO/PM6:Y6/MoO_3/Ag	0.78	22.9	59.2	10.6
EMB-9/HIL-E100/ZnO/PM6:Y6/MoO_3/Ag	0.70	17.9	29.8	3.7

Table S 2: Photovoltaic parameters of the best performed ITO-free OPVs with laser-printed and embedded Ag nps 9-line grid, using three different PEDOT:PSS formulations, PH, PH500 and HIL-E100.

Device	V_{oc} [V]	J_{sc} [mA/cm^2]	*FF* [%]	*PCE* [%]
ITO/PH/ZnO/PM6:Y6/MoO_3/Ag	0.80	19.8	54.5	8.6
ITO/HIL-E100/ZnO/PM6:Y6/MoO_3/Ag	0.78	22.9	59.2	10.6
ITO/PH500/ZnO/PM6:Y6/MoO_3/Ag	0.76	26.8	59.6	12.1
EMB-9/PH/ZnO/PM6:Y6/MoO_3/Ag	0.76	7.5	34.3	1.9
EMB-9/HIL-E100/ZnO/PM6:Y6/MoO_3/Ag	0.76	16.8	37.1	4.7
EMB-9/PH500/ZnO/PM6:Y6/MoO_3/Ag	0.76	22.5	52.5	8.9

Figure Captions

Figure S 1. Investigation of ITO-free inverted OPV devices using the as printed not embedded Ag nps 9-line grid, a) cross-section profiles of the as laser-printed Ag nps grids on glass substrate, together with cross-section profiles after the coating of each functional layer. b) Illuminated *JV* characteristics of best performed ITO-based and ITO-free inverted OPVs using 700nm thick PEDOT:PSS HIL-E100 formulation.

Figure S 2. Atomic force microscopy images of Ag nps grid, a) before and b) after embedding process.

Figure S 3. Illuminated *JV* characteristics of the best performed ITO-free OPV devices, using embedded Ag nps 9-line (EMB-9) grid, with three different PEDOT:PSS formulations, PH, PH500 and HIL-E100.

Figure S 4: Profilometric analysis of the LIFT-printed and laser sintered Ag nps grid, a) as-printed, b) as-embedded and coated with PEDOT:PSS PH500 ~40nm layer followed by annealing at 140°C – 20 minutes.

Figure S 5. Photocurrent mapping images of 9 mm^2 inverted OPV devices, ITO-based reference ITO-free OPV devices, with different number of laser-printed parallel line embedded Ag grids. The reference device in that case was ITO/PH500/ZnO/PM6:Y6/MoO_3/Ag.

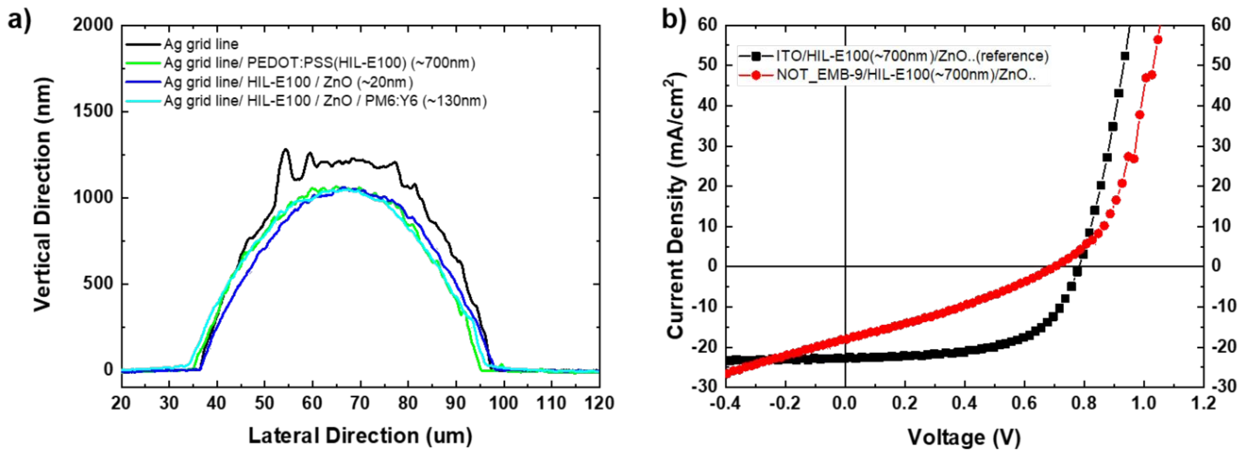

Figure S 1

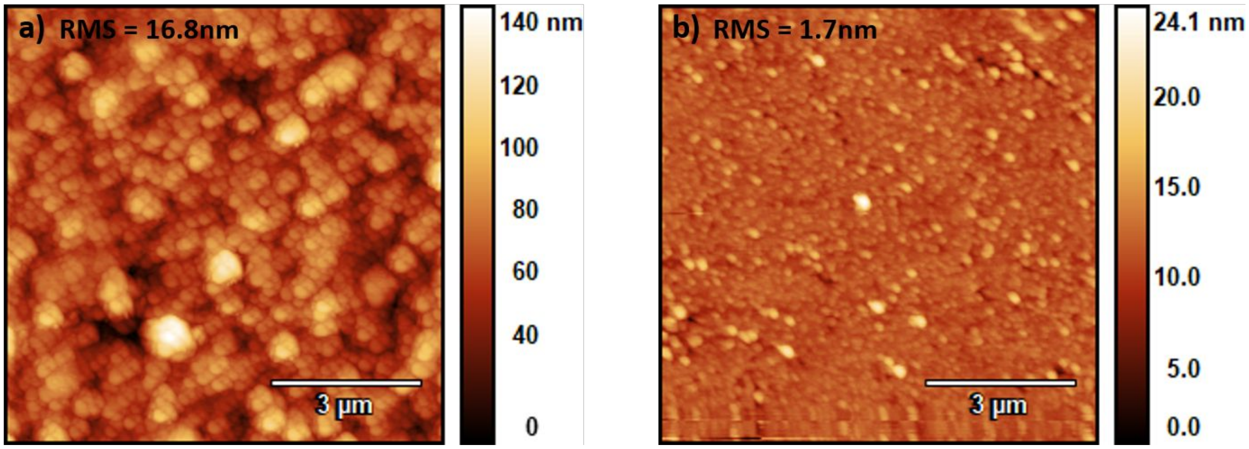

Figure S 2

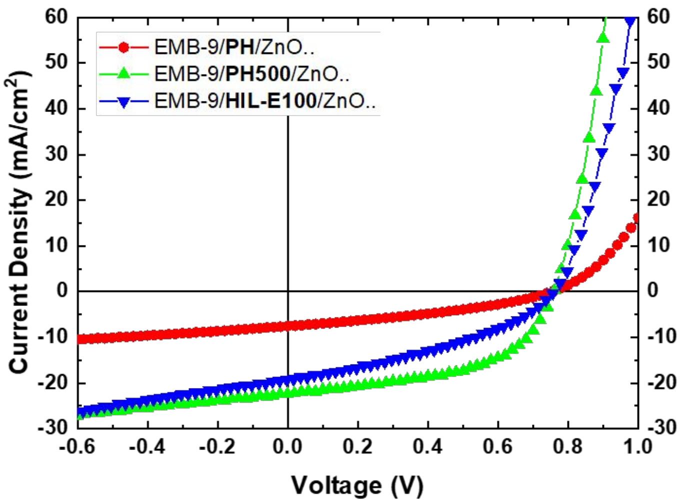
EMB-9/PH/ZnO..
EMB-9/PH500/ZnO..
EMB-9/HIL-E100/ZnO..
Current Density (mA/cm2)
Voltage (V)
60
50
40
30
20
10
0
-10
-20
-30
-0.6
-0.4
-0.2
0.0
0.2
0.4
0.6
0.8
1.0

Figure S 3

a)

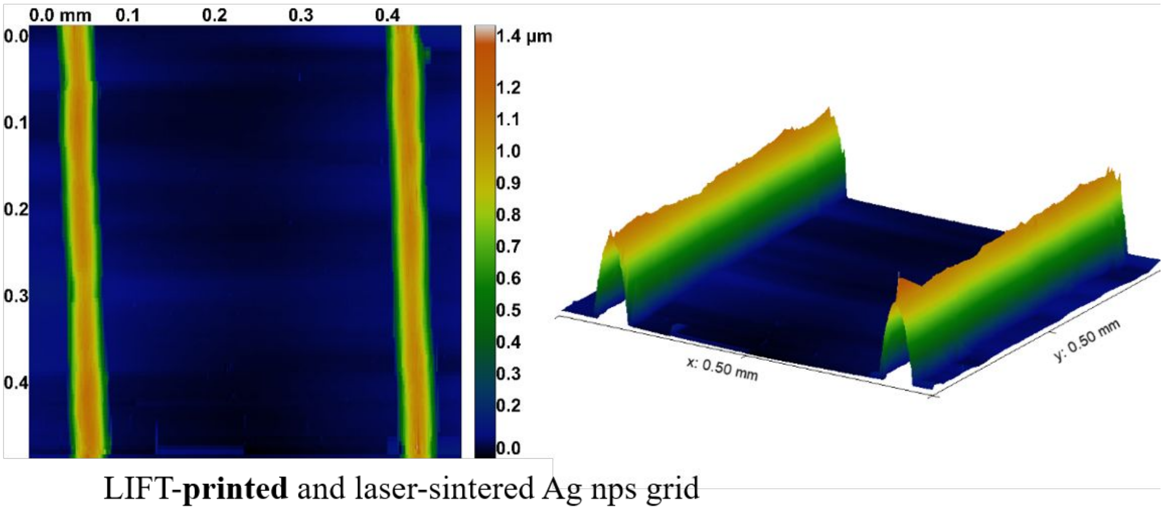

LIFT-**printed** and laser-sintered Ag nps grid

b)

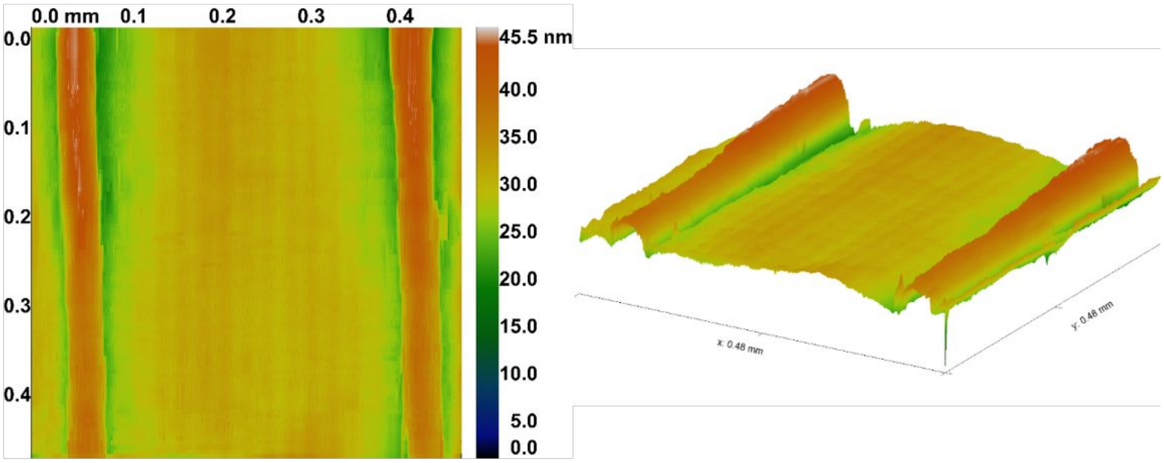

LIFT-printed and laser-sintered and **EMBEDDED** Ag nps grid/**PH500 (~ 40nm)** - **annealed**

Figure S 4

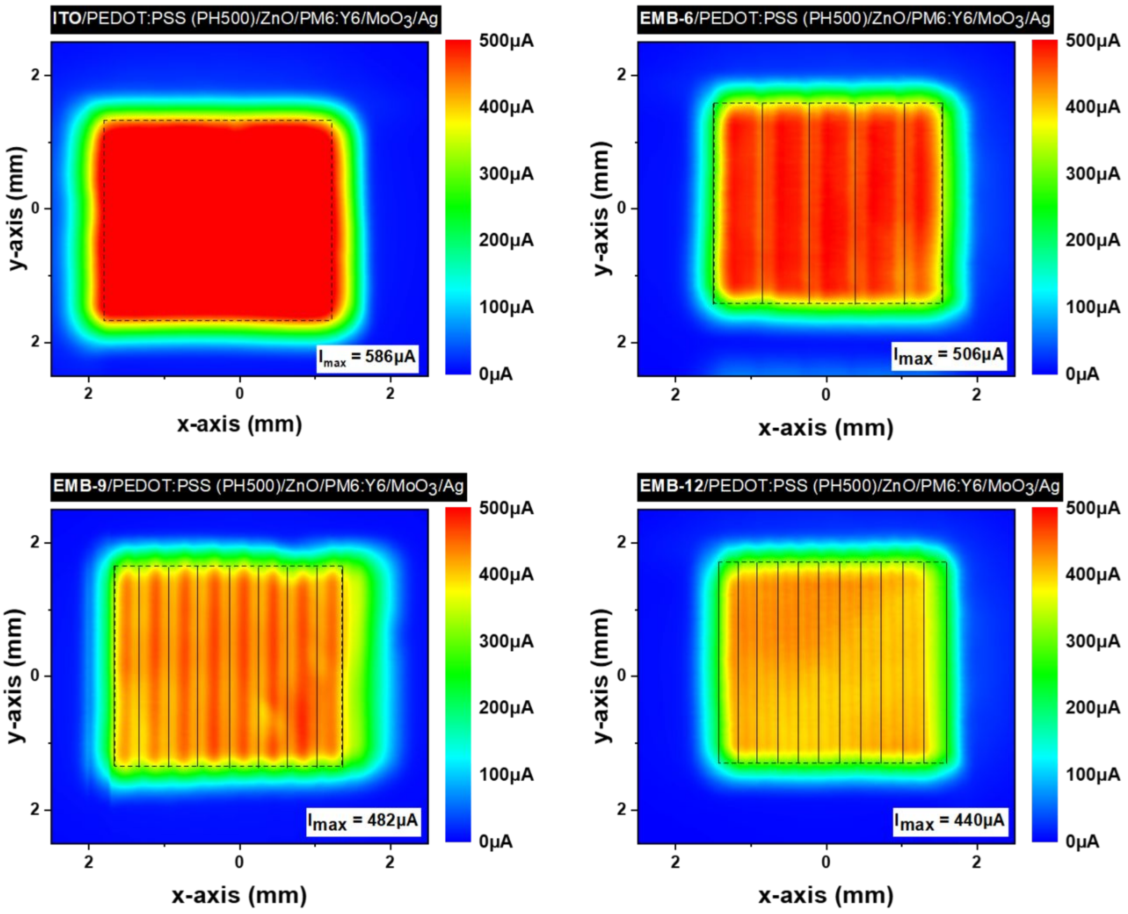
ITO/PEDOT:PSS (PH500)/ZnO/PM6:Y6/MoO3/Ag
EMB-6/PEDOT:PSS (PH500)/ZnO/PM6:Y6/MoO3/Ag
EMB-9/PEDOT:PSS (PH500)/ZnO/PM6:Y6/MoO3/Ag
EMB-12/PEDOT:PSS (PH500)/ZnO/PM6:Y6/MoO3/Ag
Imax = 586µA
Imax = 506µA
Imax = 482µA
Imax = 440µA
500µA
400µA
300µA
200µA
100µA
0µA
x-axis (mm)
y-axis (mm)

Figure S 5
